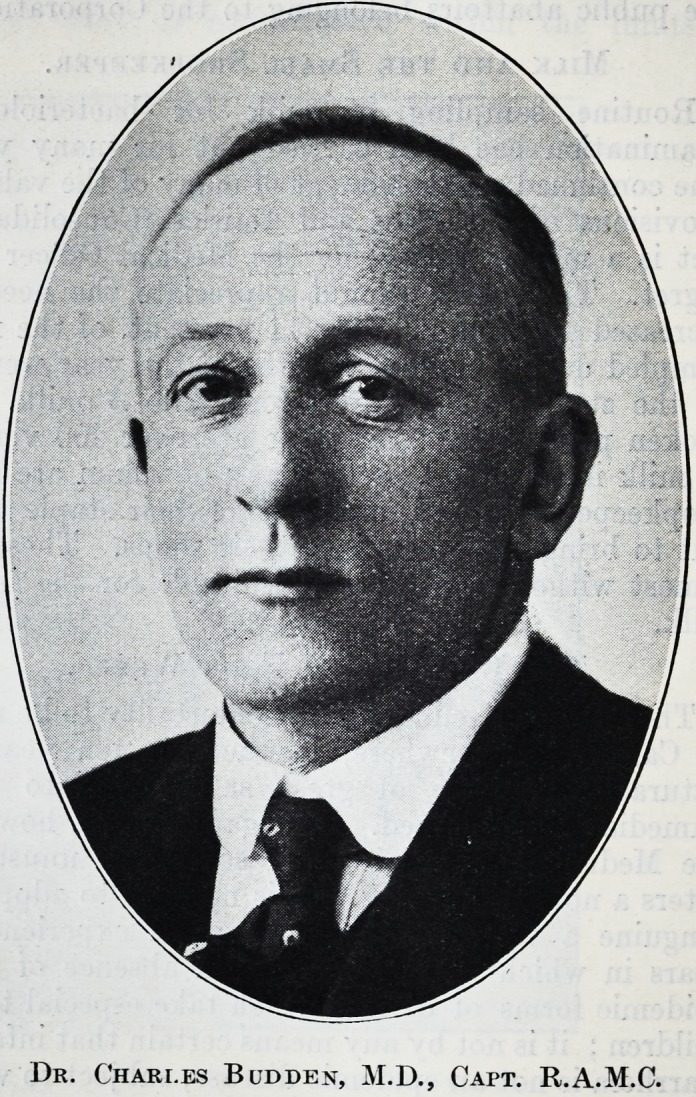# Our Health Week Competition: The Winning Papers

**Published:** 1924-05

**Authors:** 


### OUR "HEALTH WEEK" COMPETITION.

### A CLOSE CONTEST.

### THE PRIZE DIVIDED.—FOUR CONSOLATION PRIZES.

The competition for the prize of £100 offered by "The Hospital and Health Review" for the best account of how to organise a "Health Week," which was first announced in our January number, has been a remarkable success. The number of competitors was almost unexpectedly large, and included every class of person more or less closely concerned professionally with the public health—Medical Officers, other medical men, hospital secretaries, Health Visitors, clerks to Insurance Committees, sanitary inspectors, and nurses, with a sprinkling of the general public to whom the subject makes an especial appeal. The bulk of the papers reached a high level of excellence. The details had been carefully considered and were set out with considerable skill and a clear appreciation of the imperative need of so arranging the programme of a "Health Week" that it shall appeal to all classes and ages. With so large a mass of excellent material before them the task of the adjudicators was full of difficulties. A variety of considerations had to be kept in view, and, in the end, it was decided that the fairest course would be to divide the prize between the two competitors who made the nearest approach to fulfilling the conditions and to presenting a comprehensive and practical scheme of procedure of obvious value to promoters of "Health Weeks" throughout the country. The authors of the two premiated papers, in the alphabetical order of their surnames, are :—

Charles W. Budden, M.D., Captain R.A.M.C., 1 Market Street, Hoylake, Cheshire.

W. Allen Daley, M.D., B.Sc., D.P.H., Medical Officer of Health, Blackburn.

To each of these a cheque for £50 has been sent.

Dr. W. Allen Daley, M.D., B.Sc., M.O.H. of Blackburn

Dr. Charles Budden, M.D., Capt. R.A.M.C.

Dr. Budden has achieved distinction both as a medical man and a writer. He is M.B. and Ch.B. of Victoria University, Manchester, and an M.D. of Liverpool University, and is Honorary Lecturer of the Hoylake Day Nursery. As a Territorial Captain in the Royal Army Medical Corps he was in charge of the Dysentery Section of the British Salonica Force. He is well known as a writer and lecturer. He has written and lectured upon "The Beauty and Interest of Wirral," "Rambling Round the Old Wirral Churches" (Wirral is a famous Cheshire peninsula), "Pictures in Music," "The Seven Lamps of Music," and "Model Making for the Sunday School." His most ambitious literary work is, however, "The Local Colour of the Bible" (3 vols.) in collaboration with the Rev. Edward Hastings. His distinctively professional writings are "The Way of Health," a dissertation on "Diazo Reaction in Tuberculosis" in the "British Medical Journal," and on "Ionic Medication" in "The Practitioner." Dr. Budden, whose interest in architecture has procured for him the Honorary Associateship of the

Liverpool Architectural Society, is married to an M.D. of Glasgow.

DR. DALEY.

Dr. Daley has had a distinguished academic career, graduating B.A. from the Royal University of Ireland with honours in 1906. In the same year he took the B.Sc. of London and four years later the M.B. and B.S. (Honours Med.), and in 1912 the M.D. At Liverpool, where he graduated M.B. and Ch.B., he won numerous prizes—the Kanthack Medal in Pathology, the George Holt Medal in Physiology and the Robert Gee Prize for Diseases of Children. His D.P.H. he took at Cambridge with distinction. Dr. Daley has been House Physician at the Royal Infirmary, Liverpool, House Physician and Senior Resident Medical Officer at Liverpool Children's Infirmary, and was Civil Medical Officer of the Dock Battalion of the King's Liverpool Regiment, and M.O.H. at Bootle. A particularly active Medical Officer, Dr. Daley, who is a Fellow of the Royal Society of Medicine, is also a frequent contributor to the medical press. He has written on "Rats and their Extermination," "Costs and Results of Various Public Health Activities," and "Atmospheric Pollution and Health."

FURTHER AWARDS.

When they had selected the two obviously best papers, the task of the adjudicators was by no means finished. There remained so many suggestive and well-written ones that justice seemed to require that they should receive some distinctive recognition. It was therefore decided to enlarge the offered rewards, to give four Consolation Prizes of £5 each, and to make a number of Highly Commended and Commended mentions. The lists of the three classes are as follows :—

CONSOLATION PRIZES. £5 EACH.

Miss COOPER-HODGSON, 48 Old Elvet, Durham.
Mr. T. CREW, 144 London Road, Leicester.
Dr. CLAUDE LILLINGSTON, South View, Elm Grove Road, Gorleston.
Dr. C. S. THOMSON, D.P.H., M.O.H. for Deptford, 61 Wickham Road, Brockley, S.E. 4.

HIGHLY COMMENDED.

Miss IDA BATTY, Whinmead, Prestwood, Gt. Missenden.
Mr. C. E. CHAPMAN, Gravesend Hospital, Kent.
Mr. J. C. LEE GORDON, The Pines, Tile Hill, Coventry.
Dr. AUGUSTUS GRIFFITH, Town Hall, Hove.
Miss E. A. HANCOX, 334 Glossop Road, Sheffield.
Mr. A. HODGKINSON, 6 Elm Street, Corbridge, Stoke-on-Trent.
Mrs. LEACH, 16 Dale Street, Belfield, Rochdale.
Dr. NORMAN MEACHEN, Marston, Crowstone Road North, Westcliff.

COMMENDED.

Miss E. L. GARD, 10 Hampstead Hill Gardens, N.W. 3.
Miss E. N. HILL, Rose Cottage, River, Dover.
Miss A. R. MEEK, 6 Monument Retreat, Waterworks Road, Edgbaston.

IMPRESSIONS THAT REMAIN..

The dominating impressions remaining from the perusal of the piles of papers through which the adjudicators patiently worked their way are how big a thing a "Health Week" may be if properly organised, and how desirable it is that such a "Week" should be held from time to time in every considerable centre of population. It was inevitable that many of the papers should bear some family resemblance, since there are certain features, such as exhibitions and lectures, which necessarily accompany any "Health Week" worthy of the name. A few competitors, apparently not clearly understanding what was desired, wrote mere essays on the desirability of inculcating interest in the public health, or expressed their personal opinions on highly controverted subjects such as alcohol and tobacco. One competitor suggested that work on the "Week" should be started a year ahead; others desired prizes to be given for the best "Health Joke" and the best "Slogan." Yet others proposed that there should be a paid organiser, and that the local hospitals should be thrown open to visitors during the "Week." One competitor's paper was in verse, and some of the others were of rather formidable length, notably one which would have filled six or seven pages of "The Hospital and Health Review."

---

### THE WINNING PAPERS.

**By CHARLES W. BUDDEN, M.D., of Hoylake, Cheshire.**

CALLING TOGETHER A REPRESENTATIVE COMMITTEE.

In districts where a "Welfare Committee" is in being, such a committee should form the basis of organisation. The following bodies or class representatives should be included :—1. The Educational Authorities (at least one member). 2. Anglican Clergy, Roman Catholic Priests, Nonconformist Ministers. 3. Doctors. 4. Town or District Council (at least one member). 5. Hospital Committee (ditto). 6. Red Cross (ditto). 7. Girl Guides, Boy Scouts and similar organisations (ditto). 8. Any other important organisation connected with the town or district whose work is clearly in harmony with the aims of the "Health Week." If no Welfare Committee exists, someone must act as Convener of the first meeting. His or her position can then be made official at that meeting if it should see fit to constitute itself a regular body.

THE INAUGURAL COMMITTEE MEETING

1. Nominate a Chairman. Do not let this vital step be an arbitrary choice at the moment. Much will depend upon the chair. A chairman can be excessively irritating by his or her want of technique in the handling of a meeting, and can cause exasperating and even fatal delays. It is a bad plan to choose a person simply because he has money. Efficiency and a sense of humour are essential. All officers should be arranged for well ahead, and not be left to the caprice of an ill-considered nomination. 2. Prepare a resolution to place before the meeting, setting forth the desirability of having a "Health Week." See that the mover and seconder of the resolution are prepared to lay before the committee in a few words cogent reasons why such a week should be organised. 3. The committee approving broadly of the idea, move that a town's meeting be summoned in order to lay before the public a full description of a " Health Week " and its aims. Then appoint a sub-committee of, say, five people to arrange all details. Appoint a Convener for such committee. 4. Secure a guarantee fund to cover the costs of the meeting. 5. Nominate officers (see above) whose names will be submitted to the public meeting for election :—President, Vice-presidents, Chairman of Executive Committee, Secretary ditto, Treasurer.

### Organisation of the Public Meeting.

1. Secure the best hall in the district. 2. Approach the civic authorities and gain their sympathy and co-operation. 3. Advertise the meeting by newspaper advertisements and "puffs," by posters, handbills, etc. Secure an audience by personal canvass. See that reporters are present. 4. Secure a thoroughly representative " platform." 5. Select an efficient chairman for the meeting. He should occupy a position in the town, be a good speaker, be sympathetic towards the idea and versed in the technique of the chair. 6. Arrange small tables in the hall at which secretaries will sit, who will take the names, addresses and qualifications of those who offer help. 7. Secure telegrams of good wishes from the King and Queen. Send to them a wire respectfully asking for their gracious expression of encouragement. Let the reply be prepaid, and prepaid sufficiently to allow of a longer telegram than the standard twelve words. Our King and Queen are most considerate in their attention to such requests, and indeed must receive them with pleasure when they express an obvious loyalty. Such telegrams read at the public meeting will afford infinite encouragement and stimulus. A reply of thanks must, of course, be sent from the meeting. 8. Arrange for two good speakers, one to emphasise the importance of the " Health Week," the other to give a practical account of the proposed organisation. The first of these speakers should be a medical man, preferably the M.O.H. But the ability to address an audience is more important than the speaker's profession. 9. The meeting will need a resolution. This can be proposed and seconded by the two speakers respectively, and be put to the meeting by the chairman. 10. The officers nominated by the Committee should then be elected and the Committee itself made executive with power to co-opt other members and to form subsidiary committees. 11. Many good meetings are spoilt at the end by a wearisome procession of votes of thanks. Arrange for one good speaker to propose a " blunderbuss " vote of thanks, thanking chairman, speakers, organisers, the authorities lending the hall, the Press, the platform, etc., and put to the meeting by the speaker, and carried with acclamation. Arrange for a pianist to strike up the " National Anthem " the moment the clapping stops, thus ending on a note of exhilaration.

### THE PRINCIPAL FEATURES.

### An Exhibition.

A separate committee should be formed for the organisation of this feature of the Week. See that doctors, chemists and nurses are included. A general manager should be appointed. The exhibition should be held in some central hall lent for the purpose, and it should include the following features :—

Hospital Exhibits.—A model operating theatre, modern cots, appliances, instruments, etc. Demonstrations should be given by trained nurses.

Welfare Exhibits.—Pictures, foods, clothes, appliances, etc. Demonstrations given by trained workers.

Day Nursery Exhibits.—A model nursery, a nursery school, etc. Demonstrations given by crèche-trained nurses and teachers.

Invite First-Class Firms to Exhibit.—Firms dealing with all hospital, welfare, and nursery requirements of every possible kind.

Public Health Exhibits.—Models of dwellings, household appliances in relation to cleanliness of food and personal hygiene, the fly peril, etc.

Food Exhibits.—Arrange for chemical and bi-chemical demonstrations, etc.

Bacteriological Exhibits.—Showing spread of disease and its prevention, etc.

X-Ray Exhibits.—Showing deformities due to wrong foods, wrong foot-gear, etc.

An Attractive Tea-Room with Ample Accommodation.—The public will forgive all mistakes so long as tea is procurable. If people cannot get their tea they will not say a good word for the exhibition. The teas should be well done and the charge reasonable.

The object of an exhibition is to demonstrate the truth of the proverb " Seeing is Believing." A thing seen and handled makes a deeper impression than a thing heard. The size and scope of an exhibition must necessarily vary with the pretensions of the town or district.

### Organised Propaganda.

A town should give itself up to the purpose of the " Week." The following methods of propaganda may be attempted :—1. In every Church on Sunday, morning and evening, special sermons dealing with the vital association of moral with physical health. In every Sunday School special lessons on the same subject. 2. Arrange for special films in every cinema house. A large number of these films is available. 3. Minor processions through the streets advertising the " Week " and its aims. Tableaux on lorries make an effective appeal.

### A Pageant and Gymkhana.

This might be held on the last day of the " Week." A separate committee should organise it and a marshal be appointed for the procession, and a general manager for the sports and games. The procession should be all-embracing in its representatives, including the civic authorities, the Fire Brigade, the local band or imported bands of musicians, Girl Guides, Boy Scouts, Boys' Brigade, Church Lads' Brigade, etc., hospitals, homes, hostels, and so forth should be invited to prepare suitable tableaux.

Prizes should be given for the best "turn out." The sports and games emphasise the value of physical training and should include, in addition to ordinary competitions in sport, such features as gymnastic displays, first-aid demonstrations, stretcher-bearing and so forth. The sports would end with the prize-giving. A Baby Show might be combined with the foregoing.

### The Essentials of Success.

The real success of such a venture does not lie simply in the organisation running well so that everyone is pleased, but in the results which accrue. It would be easy to have a carnival week and the whole thing be a "flash in the pan." The work must be followed up. The object of the "Week" is not merely to emphasise certain physiological truths, but to stimulate the desire to learn, and to carry out the principles in practice. A permanent Committee of Health should be formed whose duty will be to systematise these principles. Such a committee should work hand in hand with such existing organisations as the Welfare Committee, the hospitals, the M.O.H., etc., and assist these organisations by voluntary work on the following lines:—1. Home nursing, assisting the district nurse. Those who engaged in this work would require to submit themselves to some preliminary training, just as the V.A.D. nurses prepared for their war-work. Such training could be given by doctors, hospitals, etc. 2. Lectures and classes held by doctors, nurses or other trained people. Mothers, young girls, boys entering upon mahnood, all need teaching. The civic authorities should be asked to provide facilities in the way of premises and equipment.

---

**By Dr. W. ALLEN DALEY, M.D., M.B., Ch.B., D.P.H.,**
**Medical Officer of Blackburn.**

### How to Start a Health Week.

The first step in the organisation of a Health Week is to profit by the experience of others. This can be done by studying the literature issued by the Health Week Committee of the Royal Sanitary Institute, and by obtaining the official programme and other particulars from towns where successful Health Weeks have taken place. The next is to approach the Mayor or Chairman of the local Council or Health Committee and ask him to convene a meeting of ladies and gentlemen who would be interested in the project. It is assumed that the leading spirit in the proposal is the Medical Officer of Health or someone acting on his behalf or with his full co-operation. At the meeting the objects of a Health Week should be explained; a strong Committee should be formed and an energetic Secretary appointed. The Editors of the local newspapers should be invited to the meeting and persuaded to give publicity to it. The Committee should include represensentatives of the cinema proprietors, teachers, doctors, the clergy, and of women's and other organisations interested in social betterment.

### The Principal Features.

The Committee should settle at an early date the principal features of the Week. If they decide to have a Health Exhibition as the main feature, a date must be arranged when the Exhibition selected will be in the neighbourhood, so that heavy expenses for carriage will be avoided. An excellent Exhibition can be hired from the Central Council for Infant and Child Welfare. While it deals primarily with Child Welfare, many of the exhibits, such as those on food and fresh air, apply to all ages. Cinema films for display at lectures or at local picturedromes should be booked early. Lecturers also should be engaged well ahead. The date and main features having been fixed, an approximate estimate of the cost can be prepared and a guarantee fund started; the principal, if not the only, guarantor, should be the local Health Authority. A very successful Health Week can be run on an expenditure of £100, provided the hall where the Exhibition is held can be obtained rent free.

### The Preliminaries.

Much preliminary work is necessary if success is to be assured. On all appropriate occasions for some weeks before speakers at public meetings should be asked to endeavour to mention the date and object of Health Week. Talks on health should be offered to the members of parochial, social and political organisations; Press references should be arranged. During the week before the Exhibition opens, posters should be displayed, window bills should be sent to mills, factories and tradesmen, and leaflets or the official programme should be distributed house to house by Boy Scouts. The tramways manager will probably allow cards to be placed in the tramcars. The Health Visitors should be asked to give an address on Health Week at the Child Welfare Centres. The teachers should be asked to give a lesson on the importance of health, and to tell the children that their parents should be persuaded to attend the Exhibition and lectures. The older children should be asked to design health posters for display at the Exhibition. They should illustrate such health maxims as "Early to Bed," "Eat Fruit and Vegetables," "Have a Bath Daily." During this period local matter, especially graphic statistics and models and photographs of local institutions, should be prepared, and school children should be taught to perform one or two of the health plays which have recently been published. Advertisements in the Press and at the picturedromes will complete the preparation of the right atmosphere in which the Health Week will commence.

### The Programme.

An illustrated programme of what is to be seen each day should be issued and should include particulars of open days at clinics, special schools, Child Welfare Centres, Hospitals, etc. If the distribution of a large number of copies can be guaranteed the advertisements should pay for it. The programme should contain a list of the churches where on the Sunday there will be sermons on Health Week. This can be arranged through the various religious leaders. On Monday, streamers containing such wording as "Blanktown Health Week," "Go to Health Exhibition," should be strung across the main streets. The principal inhabitants of the town should be invited to the opening of the Exhibition.

There should be a few brief speeches. School children should perform a health playlet and good attendance certificates should be presented to mothers who attend the Child Welfare Centres. Prizes should be presented to those mothers who have done the best in the sewing and cookery classes held at the Schools for Mothers. Local picturedrome proprietors should be asked to take a film of the opening and show it during the week at all the cinemas. Arrangements should be made for all school children over ten years of age to attend the Exhibition in groups and to write essays on what they saw. Books or other prizes should be given for the best essays.

### Many Activities.

Each afternoon and evening lantern lectures should be given in a hall adjacent to the Exhibition. The Health Week Committee should offer to send a speaker to the Rotary Club's Lunch held during the week. A rally at the Exhibition Hall of Boy Scouts and Girl Guides should be arranged and an address on Health given to them. Articles on Health should be sent to the papers every day : one at least should deal with the economic aspect of Health work and with the cost to the community of ill-health. Arrangements should be made for a talk on Health to be broadcasted in the course of the week. It should be impossible for anyone to be in the town for more than a few minutes without seeing something—a poster, a window bill, a tramcar notice or a street streamer—which will tell him that this is Health Week. Health will be on everyone's lips, and some at least of the lessons taught will sink home.

### The Exhibition as Nucleus.

The principal feature of the Health Week outlined above is the Health Exhibition. This is the nucleus around which any other activity can be developed—a perambulator parade, a lecture campaign, a demonstration of physical exercises or anything else desired. The Exhibition is the centre of Health Week work and can be visited any afternoon or evening. The mornings should be reserved for school children. The Exhibition teaches the eye ; questions are invited and can be answered by the stewards. Opening ceremonies and lectures take place, and these receive Press publicity. Leaflets on how to keep well are given to those desiring them.

### Health Films.

The other important feature of the week should be the showing of health films at the picturedromes. If approached tactfully most cinema proprietors will show these free of charge and will allow a speaker from three to five minutes in which to speak on Health Week. This reaches in the course of the week a very large audience.

### Insuring Success.

Two words suffice to answer the question—" How Would You Insure the Success of Your Health Week ? " They are—" Hard Work ! "

## Figures and Tables

**Figure f1:**
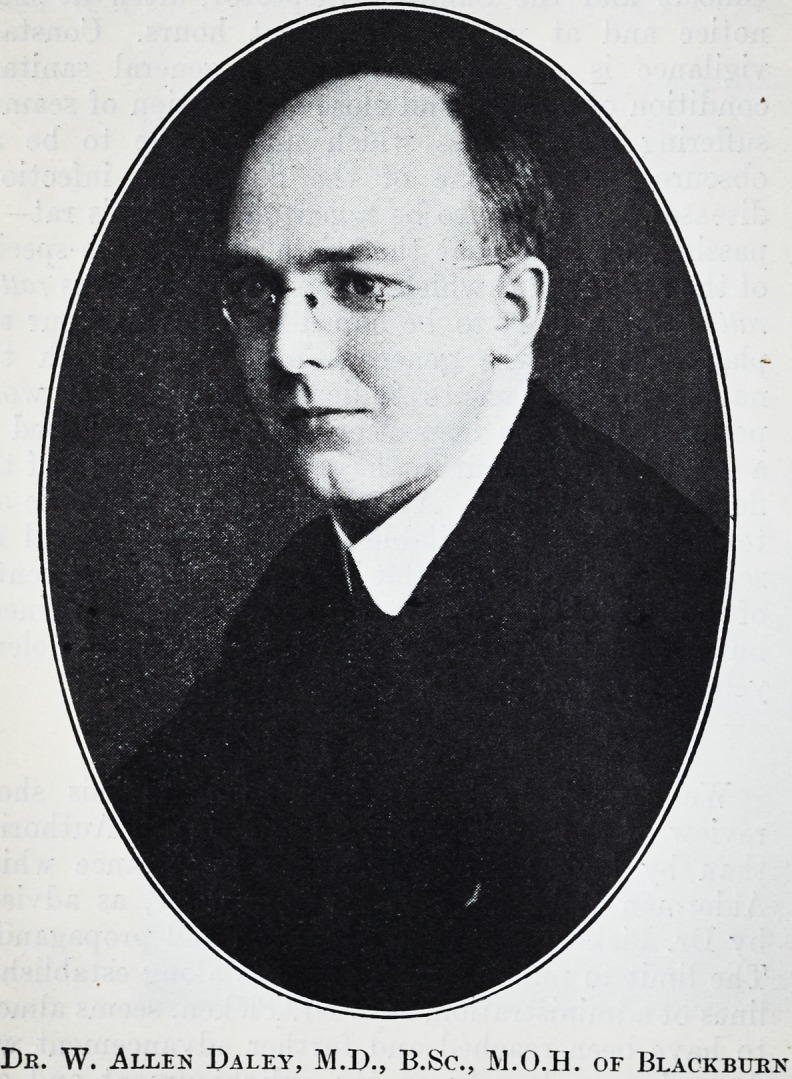


**Figure f2:**